# Unveiling the role of ferroptosis in the progression from NAFLD to NASH: recent advances in mechanistic understanding

**DOI:** 10.3389/fendo.2024.1431652

**Published:** 2024-07-04

**Authors:** Qian Yu, Lixing Song

**Affiliations:** Laboratory Medical Department, Zigong Fourth People’s Hospital, Zigong, China

**Keywords:** NAFLD, NASH, lipid peroxidation, pathogenesis, ferroptosis

## Abstract

Non-alcoholic fatty liver disease (NAFLD) is a prevalent and significant global public health issue. Nonalcoholic steatohepatitis (NASH) represents an advanced stage of NAFLD in terms of pathology. However, the intricate mechanisms underlying the progression from NAFLD to NASH remain elusive. Ferroptosis, characterized by iron-dependent cell death and distinguished from other forms of cell death based on morphological, biochemical, and genetic criteria, has emerged as a potential participant with a pivotal role in driving NAFLD progression. Nevertheless, its precise mechanism remains poorly elucidated. In this review article, we comprehensively summarize the pathogenesis of NAFLD/NASH and ferroptosis while highlighting recent advances in understanding the mechanistic involvement of ferroptosis in NAFLD/NASH.

## 1 Introduction

Over the past few decades, NAFLD has emerged as the most prevalent chronic liver disorder and a leading cause of death and morbidity worldwide, garnering significant attention from hepatologists and metabolism experts. NAFLD can progress to a more severe form called NASH, which is associated with advanced liver diseases like cirrhosis and hepatocellular carcinoma, ultimately resulting in mortality (1). Therefore, preventing the progression from NAFLD to NASH is crucial for reducing mortality rates. Although systematic studies have established a close bidirectional association between NAFLD and metabolic disorders such as obesity, insulin resistance, and abnormal iron homeostasis (2–4), the precise mechanisms underlying this progression remain to be explored.

Ferroptosis was proposed by Dixon in 2012 and has since been extensively studied in various pathologies including neurodegenerative diseases, tumors, and endocrine disorders (5–7). Compared to other forms of cell death such as apoptosis, autophagy, or necrosis; ferroptosis exhibits distinct genetic, morphological, and biochemical features (5). For instance, it involves iron-dependent peroxidation of lipids rather than being mediated by specific protein effectors like pore-forming proteins (8). The etiology of ferroptosis has been elucidated through various pathways including cysteine/glutathione (GSH)/glutathione peroxidase 4 (GPX4), ferroptosis suppressor protein 1 (FSP1)/coenzyme Q (CoQ), lipid peroxidation and iron overload (9).

Recently, accumulating evidence strongly suggests that ferroptosis plays a significant role in the pathogenesis of NAFLD. For instance, the liver serves as a crucial iron reservoir and is involved in various aspects of iron metabolism, including absorption, utilization, storage, and secretion (10). Studies have indicated that NAFLD patients exhibit elevated serum ferritin levels and serum ferritin levels are associated with histologic severity and advanced fibrosis in NAFLD patients (11, 12).Consistent with this, excessive iron accumulation can contribute to hepatocyte fibrosis (13, 14). Additionally, ferroptosis has been shown to induce oxidative stress, establishing a link between ferroptosis and NAFLD (15). Therefore, an updated review elucidating the role and mechanism of ferroptosis in the progression from NAFLD to NASH is imperative for enhancing our understanding of this disease and controlling mortality rates. In this review article, we provide a concise summary of the pathogenesis underlying NAFLD/NASH and discuss recent advancements regarding the interplay between ferroptosis and NAFLD/NASH.

## 2 Pathogenesis of NAFLD

As a prominent cause of chronic liver disease globally, NAFLD is not solely a metabolic disorder but encompasses a spectrum of conditions ranging from non-progressive patterns (nonalcoholic fatty liver, NAFL) to progressive patterns (NASH), ultimately leading to cirrhosis and liver carcinoma. Pathologically, it is characterized by the presence of steatosis in more than 5% hepatocytes without excessive alcohol consumption or other chronic liver diseases (16). Although the precise mechanism underlying the progression from NAFL to NASH remains elusive, numerous studies have demonstrated initiating factors including an imbalance of fat metabolism, insulin resistance, cellular stress, immune infiltration and inflammatory reactions (2, 17–20).

### 2.1 Fat metabolism in NAFLD

**Figure 1:**
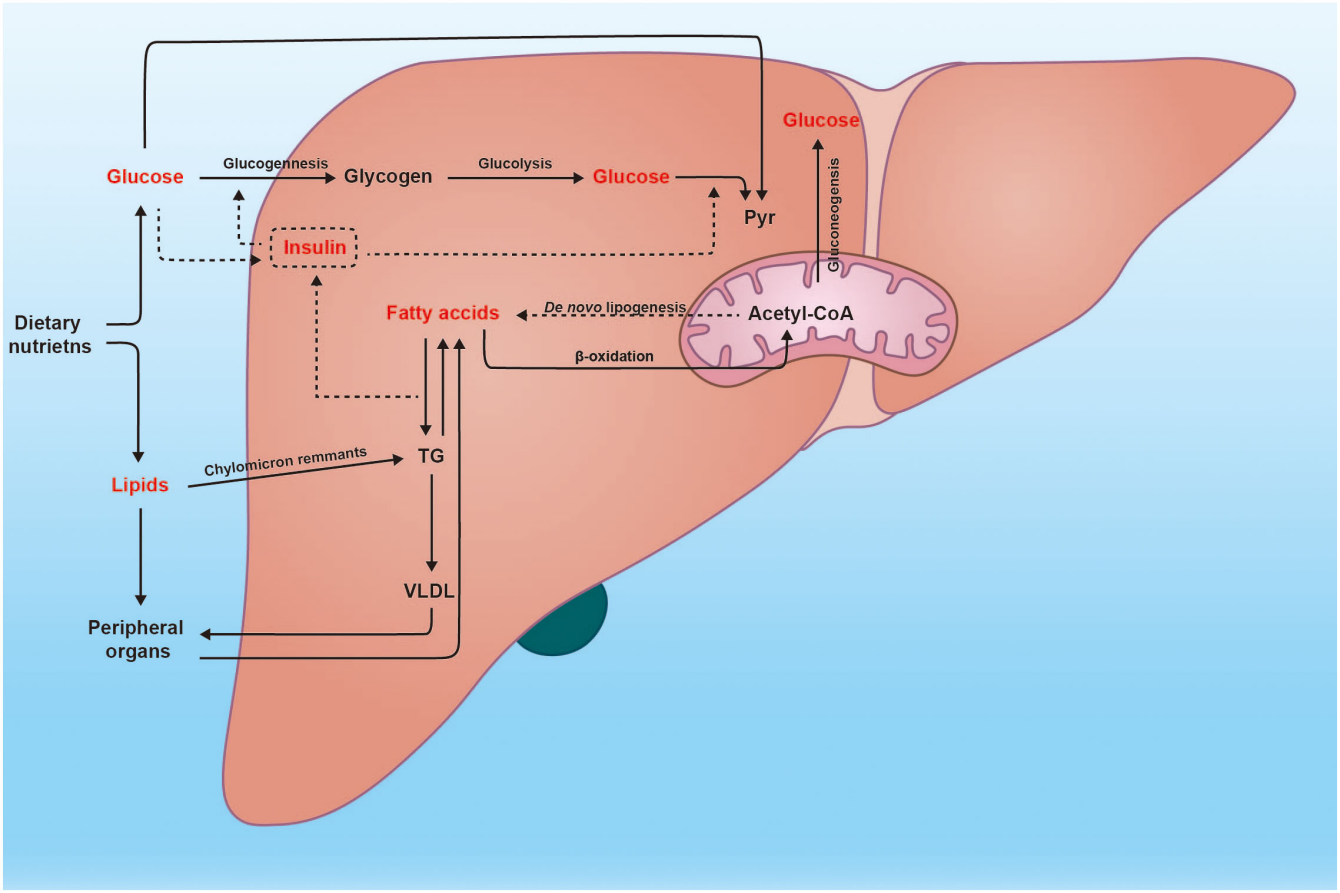
Glucose and lipid metabolism in the liver. The liver plays a crucial role in numerous physiological processes, particularly in metabolism. Subsequent to a meal, the heightened glucose levels trigger insulin secretion, thereby facilitating metabolic processes such as glycogenesis, glycolysis, and fatty acid synthesis. Moreover, excessive glucose can stimulate *de novo* lipogenesis within the liver. Conversely, fatty acids can also be converted into glucose through the gluconeogenesis pathway. Additionally, the liver is responsible for assembling very low-density lipoprotein (VLDL) and transporting endogenous triglycerides to peripheral organs.

Overnutrition, characterized by excessive consumption of any food, can disrupt liver energy metabolism balance. In this context, the intake of carbohydrates (mono-, di- and polysaccharides) and fat exceeds the body’s expenditure, resulting in a net accumulation of energy as triglycerides in the liver and white adipose tissues (WAT), which can account for the NAFLD in obese individuals (21). Additionally, apart from lipolysis of absorbed fat in the intestine, augmented hepatic *de novo* lipogenesis (DNL) fueled by excess glucose also leads to an increased flux of free fatty acids (FFAs) into the liver (**Figure 1**). Moreover, expansion of adipose tissue reservoirs in both the liver and WAT creates a susceptible environment where macrophages infiltrate and become activated due to various stimuli such as endotoxins, adipokines, lipids, and lipid metabolites (22). Subsequently, this proinflammatory state promotes insulin resistance—a defect associated with metabolic syndrome and type 2 diabetes—thus accelerating NAFLD progression through sustained delivery of fatty acids to the liver (23). Interestinglyenough, dyslipidemia resulting from irregular dietary habits can contribute to insulin resistance; conversely, insulin dysfunction caused by other factors can also lead to abnormal adipose metabolism (24, 25). Although NAFLD prevalence among obese individuals is reported up to 75.27%; another study has demonstrated that within the NAFLD population approximately 40.8% were non-obese individuals who eventually accounted for 19.2% lean subjects (26, 27). The plausible mechanism underlying this phenomenon involves impaired storage capacity within subcutaneous adipose tissue forcing fat migration towards visceral adipose tissues and ultimately leading to insulin resistance and NAFLD (28).

### 2.2 Insulin resistance in NAFLD

Insulin resistance is a pivotal factor underlying the progression from NAFL to NASH. As the master regulator of hepatic glucose and adipose metabolism, insulin secreted to the portal vein exhibits two-to threefold higher concentrations than peripheral plasma (29). It exerts multiple crucial biological effects that maintain homeostasis and prevent dysregulation (30). Following a meal, increased insulin levels directly activate insulin receptor tyrosine kinase, leading to reduced hepatic glucose concentration through enhanced glucose uptake and consumption in tissue cells (31). This process accelerates hepatic glycogen synthesis while inhibiting gluconeogenesis, thereby decreasing DNL in the liver (31). In addition to this direct mechanism of transcriptional regulation in hepatic gluconeogenesis, there exists a faster indirect mechanism involving the reduction of peripheral white adipose tissue (WAT) lipolysis through IRTK/AKT2 pathway (29). This change acutely influences hepatic acetyl-CoA content and pyruvate carboxylase activity via substrate push and allosteric mechanisms (23). Furthermore, decreased peripheral WAT lipolysis also impacts the flux of fatty acids into the liver as substrates for triglyceride synthesis (32).

### 2.3 Oxidative stress and cell death in NAFLD

The imbalance of adipose metabolism also creates a lipotoxic microenvironment, wherein the increased oxidation of free fatty acids generates an excessive production of reactive oxygen species (ROS), which serves as a key driver of cellular stress, including oxidative stress and endoplasmic reticulum (ER) stress (18). Subsequently, ROS-induced oxidative stress can activate a series of inflammatory reactions through the nuclear factor-κB (NF-κB) pathway and mitogen-activated protein kinases (MAPK) pathway (33, 34). Moreover, the elevated ROS production not only leads to ER stress but is reciprocally influenced by it, thereby establishing a detrimental cycle (35). Additionally, ROS can stimulate hepatic stellate cells (HSCs) to produce collagen type I via NADPH oxidase (NOX), which is considered a crucial biomarker for fibrogenesis (36). These disruptions in internal environment homeostasis trigger complex and interdependent inflammatory and immune signaling pathways that ultimately result in hepatocyte injury characterized by hepatocyte ballooning and cell death - key histological features of NASH (37). Although hepatocyte ballooning represents specialized cellular degeneration rather than cell death per se, it can still enhance pericellular inflammation and fibrotic signaling (38). Mechanisms underlying hepatocyte death primarily involve apoptosis, necroptosis, and pyroptosis. Apoptosis is programmed cell death mediated by various factors such as apoptosis signaling-regulated kinase 1 (ASK-1), caspase, and FADD-like apoptosis regulator (39–41). In contrast to apoptotic mechanisms that do not influence pericellular inflammation or damage significantly, both necroptosis and pyroptosis are pathological forms of cell death associated with cell swelling and membrane rupture leading to the release of inflammatory signals (20). Respectively, necroptosis is regulated by a cascade phosphorylation of RIPK1/RIPK3, while pyroptosis relies on gasdermin family proteins that create pores in the plasma membrane (42–44). These intricate interactions between cell injury, cell death, and proinflammation establish a fibrogenesis milieu where paracrine inflammatory signals from various cells (e.g., hepatocytes, hepatic stellate cells, liver sinusoidal endothelial cells, macrophages, and neutrophils) drive fibrosis by CCR2, CCL5/CCR5, CXCL10 and CCL20 (45–48).

Furthermore, emerging evidence suggests that ferroptosis is closely associated with NAFLD/NASH pathogenesis (15, 49, 50)

## 3 Pathogenesis of ferroptosis

**Figure 2:**
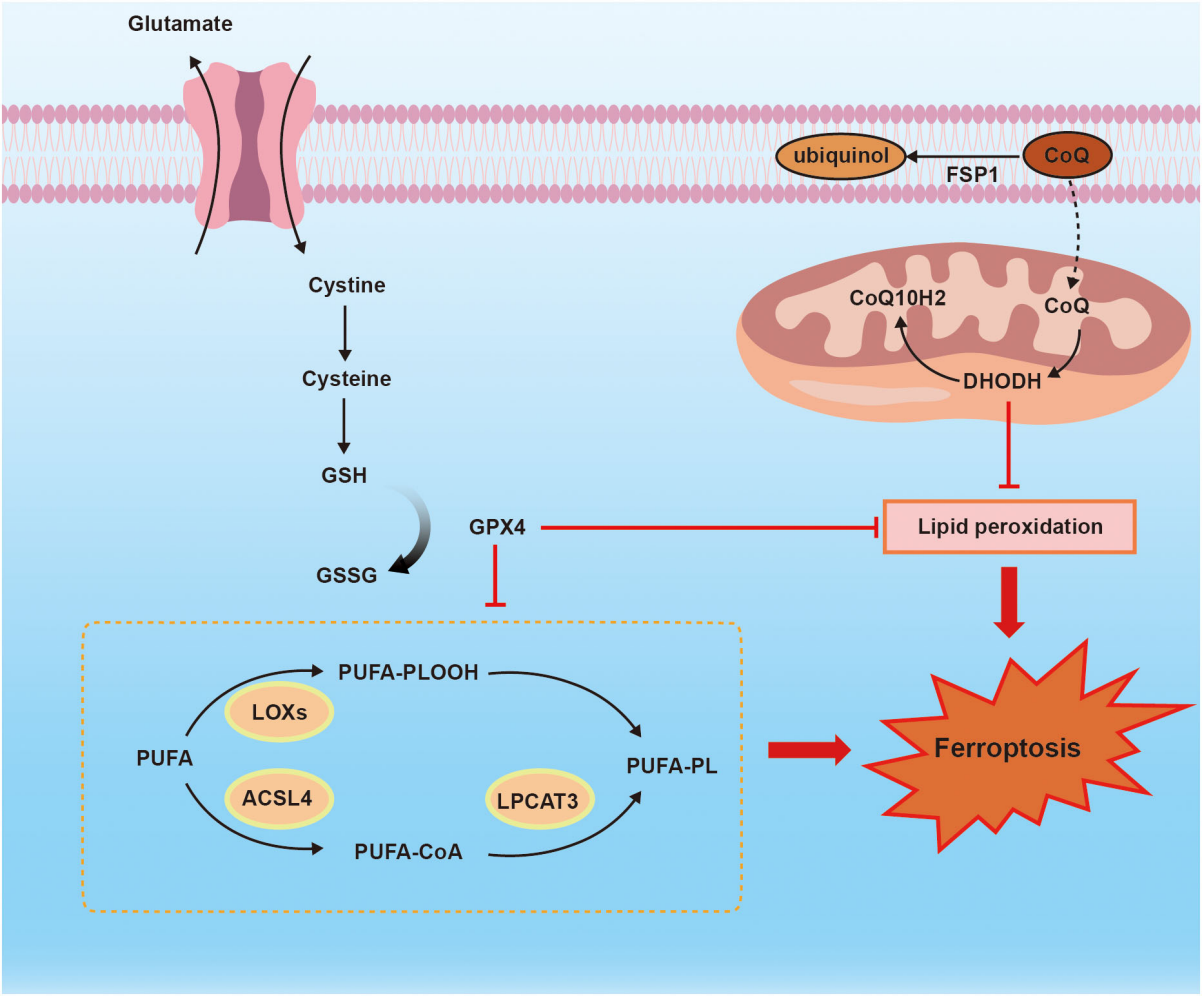
the mechanism of ferroptosis. Ferroptosis is regulated by Xc^_^/GSH/GPX4 axis, FSP1/CoQ10 axis, and PUFA-PLOOH. Membrane system xc- mediates cystine uptake into cell that is followed by GSH synthesis. FSP1 functions as an NADH-dependent CoQ, and CoQ is sufficient to suppress lipid peroxidation. ACSL4 and LPCAT3 are necessary for ferroptosis to produce PUFA-PLOOH.

Ferroptosis is a distinct mode of cell death, characterized by unique morphological, biochemical, and genetic criteria, which sets it apart from apoptosis, necrosis, and autophagy (6). Multiple mechanisms have been implicated in the occurrence of ferroptosis including Xc-/GSH/GPX4 axis, FSP1/CoQ axis, GCH1/BH4 axis, lipid peroxidation and iron overload (51–53). **Figure 2** shows the mechanism of ferroptosis.

### 3.1 Xc^_^/GSH/GPX4 axis

GPX (glutathione peroxidase) is a protein superfamily that consists of eight isoforms in mammals, with only five of them (GPX 1–4 and 6) being seleno-proteins, while GPX 5,7,8 are non-seleno proteins (54). Among all the GPXs, GPX4 is the sole enzyme responsible for directly catalyzing the reduction of lipid peroxides (54). Depletion of GPX4 in mice leads to embryonic lethality, indicating its crucial role in physiology (55). Animal trials have demonstrated that knockout mice lacking GPX4 die shortly after birth due to extensive hepatocyte degeneration; however, lipophilic antioxidant vitamin E can protect hepatocytes against lipid peroxidation (56). Additionally, various diseases such as clear-cell carcinomas, gastric cancer, and diabetic nephropathy have been associated with GPX4 (57–59). Ferroptosis is associated with iron-dependent ROS, but the GPX4 enzyme can mitigate lipid peroxidation. Yang et al. have demonstrated that depletion of glutathione (GSH) can inactivate the GPX4 enzyme, leading to ferroptosis initiation (60). System Xc- is widely distributed in the phospholipid bilayer and serves as a crucial component of the cellular antioxidant system. It consists of a heterodimeric protein comprising a light chain SLC7A11 and a heavy chain SLC3A2 (61). System Xc-primarily imports cystine and exports glutamate in a 1:1 ratio, with cysteine being an essential precursor for GSH synthesis (61). GPX4 utilizes GSH as a cofactor to convert glutathione into oxidized glutathione (GSSG) while reducing cytotoxic lipid peroxides to non-toxic lipid alcohols (62). Gao et al. discovered that both glutamine or cystine starvation could induce ROS accumulation, resulting in lipid peroxidation and ferroptosis (63). Consequently, depletion of GSH can deactivate GPX4, thereby initiating ferroptosis through inhibition of system xc- or GPX4 compounds, leading to toxic lipid ROS accumulation (64, 65). In addition, using the ferroptosis inducer erastin or the GPX4 inhibitor (1S,3R)-RSL3 ultimately leads to lipid peroxidation as well (66, 67). More recently identified transcript variants of GPX4 include cytosolic (cGPX4), mitochondrial (mGPX4), and nuclear (nGPX4) (68). Among these isoforms, cGPX has been shown to inhibit ferroptosis and protect against NAFLD in mice (69). However, a newly discovered isoform named iGPX aggravates liver damage and contributes to ferroptosis under NAFLD conditions (69). Nevertheless, in some cell types or cell lines, the inhibition of GPX4 cannot induce ferroptosis, suggesting the presence of alternative mechanisms distinct from GSH-dependent pathways (70).

### 3.2 FSP1/CoQ axis in ferroptosis

FSP1 (ferroptosis suppressor protein 1) can protect against ferroptosis in the absence of GPX4 by utilizing ubiquinone (CoQ10) (70, 71). Dai et al. discovered that FSP1 inhibits ferroptotic cell death and reduces CoQ10 levels following treatment with erastin, sorafenib, and RSL3 (72). Bersuker et al. also demonstrated that myristoylated FSP1 functions as an NAD(P)H-dependent oxidoreductase in the plasma membrane to decrease CoQ10 and generate a lipophilic radical trapping antioxidant (RTA), which effectively halts lipid peroxide propagation (70). As it is well known, mitochondria produce ATP through oxidative phosphorylation via the electron transport chain. Dihydrooriotic dehydrogenase (DHODH), a CoQ10-reducing flavoprotein similar to FSP1, localizes to the mitochondria and operates parallelly with mitochondrial GPX4 to inhibit ferroptosis by reducing CoQ to CoQH2, thereby preventing lipid peroxidation (73). Additionally, the FSP1-ESCRT-III-dependent membrane repair pathway is implicated in ferroptosis (74). Excessive lipid oxidation ultimately leads to membrane rupture, and studies have shown that endosomal sorting complexes required for transport (ESCRT) mediate cell membrane remodeling and fission reactions (75). In mammalian cells, the ESCRT-III complex comprises 12 subunits known as charged multivesicular body proteins (CHMPs) (76). Among these proteins, CHMP5 and CHMP6 are crucial subunits of ESCRT-III associated with resistance against ferroptosis through membrane budding and fission; knockdown of CHMP5 or CHMP6 increases susceptibility to erastin- and RSL3-induced ferroptosis (77). Zeng et al. also observed that knockdown of FSP1 suppressed RSL3-induced expression of charged CHMP5 and CHMP6 at the plasma membrane while overexpression of CHMP5 rescued cell death induced by RSL3, erastin, and sorafenib both in wild-type cells as well as those silenced for FSP1 (76).

### 3.3 GCH1/BH4 axis in ferroptosis

Tetrahydrobiopterin (BH4) serves as a coenzyme for aromatic amino acid hydroxylases, including 5-hydroxytryptamine, dopamine, noradrenaline, adrenaline, and melatonin. Additionally, BH4 acts as a crucial cofactor for nitric oxide synthase. Exogenous administration of dopamine or melatonin has been demonstrated to suppress erastin- or hemin-induced ferroptosis in diverse cell types (78, 79). Moreover, BH4 plays a pivotal role in antioxidant defense and the removal of nitrogen oxides (80). Elevated levels of BH4 inhibit ferroptosis and confer resistance against erastin-induced cell death (81, 82). Mariluz observed that cells depleted of BH4 exhibited heightened lipid peroxidation upon GPX4 inhibition, but this effect was reversed by supplementation with BH2 (83). This finding further supports the notion that BH4 can impede ferroptosis by preventing lipid peroxidation. GTP cyclohydrolase-1 (GCH1), the rate-limiting enzyme in BH4 synthesis, selectively protects certain PUFA-PLs from degradation to alleviate oxidation and enhance resistance against ferroptosis (82). In summary, the GCH1/BH4 axis functions as a master regulator of ferroptotic resistance through its ability to block lipid peroxidation.

### 3.4 Lipid peroxidation in ferroptosis

Polyunsaturated fatty acids (PUFAs) containing bis-allylic hydrogen atoms are susceptible to lipid peroxidation, and emerging evidence suggests that PUFAs play a crucial role in the execution of ferroptosis (84). The dysregulation of lipid metabolism, characterized by increased PUFAs and decreased monounsaturated fatty acids (MUFAs), contributes to the generation of lipid peroxides, which drive ferroptosis through their incorporation into membrane phospholipids (85, 86). Kagan et al. demonstrated that hydroperoxy derivatives of polyunsaturated fatty acid-phosphatidylethanolamines (PUFA-PLs) can induce ferroptosis even in cells with GPX4 inactivation (87). Among PUFAs, arachidonic acid and adrenic acid are particularly important phospholipids involved in oxidation processes, while acyl-CoA synthetase long-chain family member (ACSL) and lysophosphatidylcholine acyltransferase 3 (LPCAT3) serve as key enzymes in lipid metabolism (88). PUFAs can undergo oxidation by ROS or LOX to form LOOHs, which subsequently generate uncontrolled lipid free radicals through Fenton-like reactions. In the presence of iron, lipid peroxides are converted into toxic lipid free radicals (LO-), leading to cellular death (8). ACSL enzymes activate free fatty acids to produce fatty acyl-CoA, which can then be incorporated into glycerophospholipids. It has been demonstrated that ACSL4 exhibits a strong preference for activating PUFAs and its deletion prevents the incorporation of PUFAs into membrane phospholipids where they would undergo oxidation following GPX4 inactivation (88). ACSL4 binds PUFAs to membrane phospholipids, creating conditions conducive for ferroptosis, while ROS directly generate PUFA-PLOOH triggering ferroptosis. In the presence of Fe2+, PUFA-PLOOH can be reduced to PUFA-PLO•, facilitating rapid chain reaction diffusion and subsequent ferroptosis (89). Furthermore, ACSL3 has also been found highly relevant to ferroptosis as it activates MUFAs that reduce plasma membrane lipids’ susceptibility to oxidation (90). Magtanong et al. further stated that ACSL3-dependent activation of MUFAs promotes a cell state resistant to ferroptosis (91).

## 4 Ferroptosis drives NAFLD to NASH

Cell death is a pivotal histologic hallmark of NASH that contributes to the amplification of inflammatory signaling and fibrogenesis. Among various forms of cell death in NASH, ferroptosis may serve as a potential trigger for initiating inflammation in steatohepatitis (92). In the early stages of NAFLD, the accumulation of lipid droplets creates a lipotoxic environment, leading to redox imbalance (93). Bioinformatics analysis revealed higher expression levels of genes involved in the “ferroptosis” pathway and “glutathione metabolism” pathway, such as ACSL6, ACSL4, GSS, GPX2, and GPX3 in individuals with NASH compared to healthy controls; meanwhile, the expression of iron exporter ferroportin (SLC40A1) was down-regulated (94). Overall, ferroptosis is closely associated with NAFLD/NASH through underlying mechanisms including iron overload, oxidative stress, and overwhelming membrane lipid peroxidation.

### 4.1 The role of iron overload between ferroptosis and NAFLD/NASH

Iron plays a diverse range of crucial roles in physiological metabolism *in vivo*. In addition to its role as an element bound to proteins for essential cellular functions such as ATP generation, DNA synthesis and repair, and oxygen transport, iron is also an indispensable component of the redox system, switching between Fe2^+^ and Fe3^+^ (95). Abnormal accumulation of iron within cells and impaired binding capacity of iron-containing proteins can result in increased levels of redox-active iron, which generates oxygen radicals through Fenton chemistry, leading to detrimental effects on DNA, proteins, and lipids (50). Nevertheless, clinical analysis has shown that elevated serum ferritin levels are independently associated with advanced NAFLD (11). This observation implies an augmentation in iron reserves and systemic inflammation within the context of NAFLD. Ferritin heavy chain (FTH) and ferritin light chain (FTL) are crucial iron-associated factors. Recently, Crawford et al. reported that reducing iron intake attenuates NAFLD in a high-calorie-induced mouse model while Deng et al. demonstrated that Caveolin-1 enhances ferritin synthesis and increases iron storage capacity by activating the FTL/FTH pathway, subsequently decreasing ROS levels and alleviating NAFLD progression (96, 97). Furthermore, Yu et al. have reported that Fe3^+^ chelation can reverse iron overload *in vivo* thereby attenuating the production of lethal ROS while alleviating ER stress and regulating the Nrf2/NF-κB signaling pathway (98). These findings propose a potential therapeutic strategy for NAFLD/NASH. Emerging evidence suggests that aberrant iron distribution contributes to a novel mechanism underlying NAFLD development; elevated secretion of iron-containing extracellular vesicles leads to hepatocyte iron deficiency which enhances lipogenesis and insulin resistance through HIF2α-ATF4 signaling whereas HSC-mediated iron overload promotes fibrogenesis via excessive ROS production (99).

### 4.2 The role of lipid peroxidation and ROS between ferroptosis and NAFLD/NASH

The pathogenesis of ferroptosis in NAFLD/NASH is closely associated with lipid peroxidation and abnormal accumulation of reactive oxygen species (ROS). For instance, in a methionine-choline deficient (MCD) diet-induced mice model, elevated arachidonic acid metabolism promotes ferroptosis, while the use of ferroptosis inhibitors alleviates inflammation, fibrogenesis, and liver injury (100). This was confirmed by measuring levels of lipid ROS and iron.

ACSL4 is a crucial enzyme that catalyzes CoA to PUFAs such as arachidonic acid, which can promote ferroptosis. Clinical studies have reported increased hepatic ASCL4 levels in NAFLD patients compared to healthy controls (101). Further research has shown that suppressing ACSL4 expression significantly improves NAFLD symptoms in multiple mouse models (101). Consistent with this finding, another study demonstrated that the ferroptosis inhibitor liproxstatin-1 (LPT1) not only reduced the expression of ACSL4 but also decreased hepatic lipids (e.g., triglycerides, cholesterol), lipid metabolites (e.g., 4-hydroxynonenal, malondialdehyde), insulin resistance, mitochondrial ROS content and liver fibrosis (102). Moreover, recent investigations on lung injury revealed that deletion of the ACSL4 gene suppresses ferroptosis and attenuates chemical-induced lung injury and pulmonary fibrosis by reducing PUFA-containing membrane phospholipids and inhibiting lipid peroxide formation (103).

The Xc-/GSH/GPX4 axis represents a central antioxidant pathway in ferroptosis. Studies have demonstrated that treatment with the ferroptosis inducer RSL-3 can decrease hepatic expression of GPX4, leading to more severe symptoms in an MCD-induced NASH mice model (66). Consistent with this, administration of the GPX4 activator sodium selenite and the ferroptosis inhibitor liproxstatin-1 can attenuate NASH severity (66). SLC7A11, a subunit of System Xc-, also plays critical roles in both ferroptosis and NAFLD/NASH pathogenesis. In high-fat diet-induced NASH mice models, hepatocyte ATF4 ablation increases susceptibility to ferroptosis and hepatocarcinogenesis; however, ectopic expression of SLC7A11 reverses these effects (104). Similarly, another study revealed that Arbutin, a natural antioxidant compound, reduces ferroptosis and ameliorates high-fat diet (HFD) induced NAFLD both *in vivo* and *in vitro* by regulating methylation of the SLC7A11 gene through inhibition of fat mass and obesity-related protein (FTO) (105).

### 4.3 Other regulatory factors between ferroptosis and NAFLD/NASH

In addition, several studies have also reported additional potential regulatory pathways linking ferroptosis and NAFLD/NASH. The key regulatory factors include PRDX3, Lgfbp7, and Mfn2. For instance, the accumulation of phospholipid peroxides can lead to hyperoxidation of peroxiredoxin 3 (PRDX3), promoting its translocation from mitochondria to the plasma membrane. This subsequently inhibits cystine uptake and induces ferroptosis (106). Recently, a study in a zebrafish model has elucidated that Lgfbp7 may regulate ferroptosis through Ncoa4-mediated ferritinophagy, with depletion of Lgfbp7 reducing hepatic iron deposition and lipid peroxidation products (107). Furthermore, Mitofusin 2 (Mfn2) could interact with inositol-requiring enzyme 1 alpha (IRE1α) to promote ferroptosis in an arsenic-induced NASH model (108).

Moreover, the pathogenic pathways are also influenced by genetic and microbiome-related factors (109). For example, Zhuge et al. proposed a possible mechanism for impaired function of antifibrotic drugs whereby gut microbiota induces lipid peroxidation and ROS accumulation to promote hepatocyte ferroptosis and activate HSCs by generating excessive chenodeoxycholic acid (110).

### 4.4 The heterogeneity of animal models on NAFLD/NASH

In this review, we have discussed four mouse models simulating the pathological process of human NAFLD/NASH, including the MCD-induced model, HFD-induced NASH model, CDE-induced model, and arsenic-induced NASH model. The first three models represent dietary intervention approaches, while the last one represents toxin exposure. However, not all models successfully replicate the etiology and metabolic features of human NASH. For example, the HFD model induces hepatic steatosis, insulin resistance, inflammation and ER stress but lacks fibrosis and NASH development; both the MCD and CDE models can induce NASH-like pathology in mice but do not exhibit insulin resistance (111). Therefore, a combination of multiple strategies such as HFD+fructose and CD+HFD is widely employed to induce insulin resistance, dyslipidemia, ER stress, fibrosis and NASH (111). Additionally, the arsenic exposure model has been reported to cause lipid accumulation, increase TNF levels, and result in liver injury (112). Furthermore, toxins or genetics plus diet-based models are also extensively used in NASH research (111).

### 4.5 The relevance between ferroptosis and NAFLD/NASH in human patients

Ferroptosis is associated with the Xc-/GSH/GPX4 axis, the FSP1/CoQ axis, the GCH1/BH4 axis, lipid peroxidation, and iron overload. Abundant clinical research has demonstrated that these mechanisms are involved in NAFLD/NASH. For example, serum ferritin levels are increased in NAFLD and hyperferritinemia is associated with advanced NASH and fibrosis (11). Consistent with this finding, another study has shown that patients with hyperferritinemia have higher iron stores and more severe liver fibrosis (113). A clinical cohort analysis reported that oral supplementation of whey protein in patients with NASH can increase plasma GSH levels, thereby reducing hepatic macrovesicular steatosis (114). Recently, novel evidence has revealed that hepatic expression of SLC7A11 is upregulated in NASH patients and positively correlates with disease severity (115). Moreover, a pathological analysis conducted on metabolic-associated fatty liver disease (MAFLD) patients demonstrated elevated levels of malondialdehyde (a marker for lipid peroxidation), while total superoxide dismutase (SOD) activity and total antioxidant capacity (TAC) were decreased in liver tissues from MAFLD patients (69). Additionally, mRNA levels of NOX1, NOX4, FTH,and FTL were also found to be higher in livers from MAFLD patients compared to controls (69). These clinical results provide incontrovertible evidence indicating the significant role of ferroptosis in the progression of NAFLD/NASH.

### 4.6 Targeting ferroptosis in NAFLD/NASH

Currently, Rezdiffra has gained approval from the Food and Drug Administration (FDA) for the treatment of NAFLD/NASH (116), marking a significant milestone in the advancement of therapeutics for this condition. In this review, we have presented various mechanisms targeting ferroptosis that can potentially mitigate or prevent the progression of NAFLD to NASH. These targeted approaches are summarized in **Table 1**. However, it is important to note that these strategies have primarily been investigated in preclinical models, and there is currently no commercially available drug specifically designed to target ferroptosis for the treatment of NAFLD/NASH.

**Table 1:** Targeting ferroptosis in NAFLD/NASH.

target	drug	mechanism	references
System Xc-	erastin	Inhabit System Xc-, leading to GSH depletion	(67)
GPX4	RSL-3	Inactivates GPX4, leading to lipid peroxidation	(66)
PUFAs	Vitamin E Liproxstatin-1 Ferrostatin-1	Inhabit lipid peroxidation	(56, 67, 102)
ACSL4	Rosiglitazone Trosiglitazone	Inhabit lipid peroxidation	(5, 101, 102)
Iron chelator	DFO DFP EWCDs (fluorescent egg white-based carbon dots)	Chelate iron, reducing lipid peroxidation	(98, 102, 117)
Caveolin-1		Alleviates ROS induced by irons	(97)
FTO/SLC7A11	Arbutin	regulating methylation of the SLC7A11 gene by inhabiting FTO	(105)

## 5 Conclusion

NAFLD/NASH has emerged as a critical public health concern, necessitating further investigation into its underlying mechanisms and treatment strategies. Conversely, the development of an accurate predictive tool for identifying individuals at risk of transitioning from NAFLD to NASH could significantly impact mortality rates. Notably, in a choline-deficient, ethionine-supplemented (CDE) diet model, inhibition of ferroptosis effectively prevents initiation of necrotic cell death and suppresses inflammatory reactions and immune cell infiltration (92). This underscore the importance of targeting ferroptosis as a potent therapeutic strategy to impede disease progression from NAFLD to NASH in the future. Although numerous animal models have provided substantial evidence demonstrating that inhibiting ferroptosis exerts significant effects on NAFLD/NASH, their precise mechanisms remain elusive. Furthermore, clinical research investigating pharmacological therapies aimed at inhibiting ferroptosis for treating NAFLD/NASH is currently lacking.

Herein, we summarize recent advances in understanding the mechanistic involvement of ferroptosis in NAFLD/NASH pathogenesis. This review article provides a novel perspective on the progression from NAFLD to NASH and these underlying mechanisms may have broader implications in other diseases such as idiopathic pulmonary fibrosis (IPF). In conclusion, ferroptosis plays a crucial role in the pathogenesis of NAFLD/NASH and offers novel therapeutic and predictive avenues for patients.

## Author contributions

QY: Writing – original draft, Writing – review & editing. lS: Visualization, Writing – review & editing.

## References

[1] Sumida Y Yoneda M. Current and future pharmacological therapies for NAFLD/NASH. J Gastroenterol. (2018) 53:362–76. doi: 10.1007/s00535-017-1415-1 PMC5847174 29247356

[2] Khan RS Bril F Cusi K Newsome PN. Modulation of insulin resistance in nonalcoholic fatty liver disease. Hepatology. (2019) 70:711–24. doi: 10.1002/hep.30429 30556145

[3] Arroyave-Ospina JC Wu Z Geng Y Moshage H. Role of oxidative stress in the pathogenesis of non-alcoholic fatty liver disease: implications for prevention and therapy. Antioxidants (Basel) 10. (2021) 26(2):174. doi: 10.3390/antiox10020174 PMC7911109 33530432

[4] Chen Z Tian R She Z Cai J Li H. Role of oxidative stress in the pathogenesis of nonalcoholic fatty liver disease. Free Radic Biol Med. (2020) 152:116–41. doi: 10.1016/j.freeradbiomed.2020.02.025 32156524

[5] Dixon SJ Lemberg KM Lamprecht MR Skouta R Zaitsev EM Gleason CE. Ferroptosis: an iron-dependent form of nonapoptotic cell death. Cell. (2012) 149:1060–72. doi: 10.1016/j.cell.2012.03.042 PMC3367386 22632970

[6] Jiang X Stockwell BR Conrad M. Ferroptosis: mechanisms, biology and role in disease. Nat Rev Mol Cell Biol. (2021) 22:266–82. doi: 10.1038/s41580-020-00324-8 PMC8142022 33495651

[7] Yan HF Zou T Tuo QZ Xu S Li H Belaidi AA. Ferroptosis: mechanisms and links with diseases. Signal transduction targeted Ther. (2021) 6:49. doi: 10.1038/s41392-020-00428-9 PMC7858612 33536413

[8] Stockwell BR Friedmann Angeli JP Bayir H Bush AI Conrad M Dixon SJ. Ferroptosis: A regulated cell death nexus linking metabolism, redox biology, and disease. Cell. (2017) 171:273–85. doi: 10.1016/j.cell.2017.09.021 PMC5685180 28985560

[9] Dixon SJ Olzmann JA. The cell biology of ferroptosis. Nat Rev Mol Cell Biol. (2024) 25(6):424–442. doi: 10.1038/s41580-024-00703-5 PMC12187608 38366038

[10] Cheng Z Chu H Zhu Q Yang L. Ferroptosis in non-alcoholic liver disease: Molecular mechanisms and therapeutic implications. Front Nutr. (2023) 10:1090338. doi: 10.3389/fnut.2023.1090338 36992907 PMC10040549

[11] Kowdley KV Belt P Wilson LA Yeh MM Neuschwander-Tetri BA Chalasani N. Serum ferritin is an independent predictor of histologic severity and advanced fibrosis in patients with nonalcoholic fatty liver disease. Hepatol (Baltimore Md.). (2012) 55:77–85. doi: 10.1002/hep.24706 PMC3245347 21953442

[12] Zelber-Sagi S Nitzan-Kaluski D Halpern Z Oren R. NAFLD and hyperinsulinemia are major determinants of serum ferritin levels. J Hepatol. (2007) 46:700–7. doi: 10.1016/j.jhep.2006.09.018 17150278

[13] Datz C Müller E Aigner E. Iron overload and non-alcoholic fatty liver disease. Minerva Endocrinol. (2017) 42:173–83. doi: 10.23736/S0391-1977.16.02565-7 27834478

[14] Handa P Morgan-Stevenson V Maliken BD Nelson JE Washington S Westerman M. Iron overload results in hepatic oxidative stress, immune cell activation, and hepatocellular ballooning injury, leading to nonalcoholic steatohepatitis in genetically obese mice. Am J Physiol Gastrointest Liver Physiol. (2016) 310:G117–27. doi: 10.1152/ajpgi.00246.2015 26564716

[15] Wang S Liu Z Geng J Li L Feng X. An overview of ferroptosis in non-alcoholic fatty liver disease. Biomedicine pharmacotherapy = Biomedecine pharmacotherapie. (2022) 153:113374. doi: 10.1016/j.biopha.2022.113374 35834990

[16] Byrne CD Targher G. NAFLD: a multisystem disease. J Hepatol. (2015) 62:S47–64. doi: 10.1016/j.jhep.2014.12.012 25920090

[17] Hodson L Gunn PJ. The regulation of hepatic fatty acid synthesis and partitioning: the effect of nutritional state. Nat Rev Endocrinol. (2019) 15:689–700. doi: 10.1038/s41574-019-0256-9 31554932

[18] Sharma S Le Guillou D Chen JY. Cellular stress in the pathogenesis of nonalcoholic steatohepatitis and liver fibrosis. Nat Rev Gastroenterol Hepatol. (2023) 20:662–78. doi: 10.1038/s41575-023-00832-w 37679454

[19] Schuster S Cabrera D Arrese M Feldstein AE. Triggering and resolution of inflammation in NASH. Nat Rev Gastroenterol Hepatol. (2018) 15:349–64. doi: 10.1038/s41575-018-0009-6 29740166

[20] Schwabe RF Luedde T. Apoptosis and necroptosis in the liver: a matter of life and death. Nat Rev Gastroenterol Hepatol. (2018) 15:738–52. doi: 10.1038/s41575-018-0065-y PMC6490680 30250076

[21] Jensen T Abdelmalek MF Sullivan S Nadeau KJ Green M Roncal C. Fructose and sugar: A major mediator of non-alcoholic fatty liver disease. J Hepatol. (2018) 68:1063–75. doi: 10.1016/j.jhep.2018.01.019 PMC5893377 29408694

[22] Kazankov K Jørgensen SMD Thomsen KL Møller HJ Vilstrup H George J. The role of macrophages in nonalcoholic fatty liver disease and nonalcoholic steatohepatitis. Nat Rev Gastroenterol Hepatol. (2019) 16:145–59. doi: 10.1038/s41575-018-0082-x 30482910

[23] Perry RJ Camporez JG Kursawe R Titchenell PM Zhang D Perry CJ. Hepatic acetyl CoA links adipose tissue inflammation to hepatic insulin resistance and type 2 diabetes. Cell. (2015) 160:745–58. doi: 10.1016/j.cell.2015.01.012 PMC4498261 25662011

[24] Joo JH Lee DW Choi DW Park EC. Association between night work and dyslipidemia in South Korean men and women: a cross-sectional study. Lipids Health Dis. (2019) 18:75. doi: 10.1186/s12944-019-1020-9 30922333 PMC6440094

[25] Wu L Parhofer KG. Diabetic dyslipidemia. Metabolism: Clin Exp. (2014) 63:1469–79. doi: 10.1016/j.metabol.2014.08.010 25242435

[26] Quek J Chan KE Wong ZY Tan C Tan B Lim WH. Global prevalence of non-alcoholic fatty liver disease and non-alcoholic steatohepatitis in the overweight and obese population: a systematic review and meta-analysis. Lancet Gastroenterol Hepatol. (2023) 8:20–30. doi: 10.1016/S2468-1253(22)00317-X 36400097

[27] Ye Q Zou B Yeo YH Li J Huang DQ Wu Y. Global prevalence, incidence, and outcomes of non-obese or lean non-alcoholic fatty liver disease: a systematic review and meta-analysis. Lancet Gastroenterol Hepatol. (2020) 5:739–52. doi: 10.1016/S2468-1253(20)30077-7 32413340

[28] Brøns C Thuesen ACB Elingaard-Larsen LO Justesen L Jensen RT Henriksen NS. Increased liver fat associates with severe metabolic perturbations in low birth weight men. Eur J Endocrinol. (2022) 186:511–21. doi: 10.1530/EJE-21-1221 35212643

[29] Petersen MC Shulman GI. Mechanisms of insulin action and insulin resistance. Physiol Rev. (2018) 98:2133–223. doi: 10.1152/physrev.00063.2017 PMC6170977 30067154

[30] Najjar SM Caprio S Gastaldelli A. Insulin clearance in health and disease. Annu Rev Physiol. (2023) 85:363–81. doi: 10.1146/annurev-physiol-031622-043133 36260807

[31] Merrins MJ Corkey BE Kibbey RG Prentki M. Metabolic cycles and signals for insulin secretion. Cell Metab. (2022) 34:947–68. doi: 10.1016/j.cmet.2022.06.003 PMC9262871 35728586

[32] Perry RJ Zhang XM Zhang D Kumashiro N Camporez JP Cline GW. Leptin reverses diabetes by suppression of the hypothalamic-pituitary-adrenal axis. Nat Med. (2014) 20:759–63. doi: 10.1038/nm.3579 PMC4344321 24929951

[33] Nakajima S Kitamura M. Bidirectional regulation of NF-κB by reactive oxygen species: a role of unfolded protein response. Free Radical Biol Med. (2013) 65:162–74. doi: 10.1016/j.freeradbiomed.2013.06.020 23792277

[34] Zhang B Yu L Zhu R Wei X Fan X Hu H. Malting barley carbon dots-mediated oxidative stress promotes insulin resistance in mice via NF-κB pathway and MAPK cascade. J nanobiotechnology. (2022) 20:331. doi: 10.1186/s12951-022-01543-1 35842638 PMC9288084

[35] Malhotra JD Kaufman RJ. Endoplasmic reticulum stress and oxidative stress: a vicious cycle or a double-edged sword? Antioxidants Redox Signaling. (2007) 9:2277–93. doi: 10.1089/ars.2007.1782 17979528

[36] Greenwel P Domínguez-Rosales JA Mavi G Rivas-Estilla AM Rojkind M. Hydrogen peroxide: a link between acetaldehyde-elicited alpha1(I) collagen gene up-regulation and oxidative stress in mouse hepatic stellate cells. Hepatol (Baltimore Md.). (2000) 31:109–16. doi: 10.1002/hep.510310118 10613735

[37] Rangwala F Guy CD Lu J Suzuki A Burchette JL Abdelmalek MF. Increased production of sonic hedgehog by ballooned hepatocytes. J Pathol. (2011) 224:401–10. doi: 10.1002/path.2888 PMC3628812 21547909

[38] Ibrahim SH Hirsova P Gores GJ. Non-alcoholic steatohepatitis pathogenesis: sublethal hepatocyte injury as a driver of liver inflammation. Gut. (2018) 67:963–72. doi: 10.1136/gutjnl-2017-315691 PMC5889737 29367207

[39] Zhang P Wang PX Zhao LP Zhang X Ji YX Zhang XJ. The deubiquitinating enzyme TNFAIP3 mediates inactivation of hepatic ASK1 and ameliorates nonalcoholic steatohepatitis. Nat Med. (2018) 24:84–94. doi: 10.1038/nm.4453 29227477

[40] Kim JY Garcia-Carbonell R Yamachika S Zhao P Dhar D Loomba R. and steatohepatitis via caspase-2 activation of S1P. Cell. (2018) 175:133–145.e15. doi: 10.1016/j.cell.2018.08.020 30220454 PMC6159928

[41] Wang PX Ji YX Zhang XJ Zhao LP Yan ZZ Zhang P. Targeting CASP8 and FADD-like apoptosis regulator ameliorates nonalcoholic steatohepatitis in mice and nonhuman primates. Nat Med. (2017) 23:439–49. doi: 10.1038/nm.4290 28218919

[42] Majdi A Aoudjehane L Ratziu V Islam T Afonso MB Conti F. Inhibition of receptor-interacting protein kinase 1 improves experimental non-alcoholic fatty liver disease. J Hepatol. (2020) 72:627–35. doi: 10.1016/j.jhep.2019.11.008 31760070

[43] Preston SP Stutz MD Allison CC Nachbur U Gouil Q Tran BM. Epigenetic silencing of RIPK3 in hepatocytes prevents MLKL-mediated necroptosis from contributing to liver pathologies. Gastroenterology. (2022) 163:1643–1657.e14. doi: 10.1053/j.gastro.2022.08.040 36037995

[44] Cao L Liu F Liu Y Liu T Wu J Zhao J. TSLP promotes asthmatic airway remodeling via p38-STAT3 signaling pathway in human lung fibroblast. Exp Lung Res. (2018) 44:288–301. doi: 10.1080/01902148.2018.1536175 30428724

[45] Seki E de Minicis S Inokuchi S Taura K Miyai K van Rooijen N. CCR2 promotes hepatic fibrosis in mice. Hepatol (Baltimore Md.). (2009) 50:185–97. doi: 10.1002/hep.22952 PMC2705470 19441102

[46] Li BH He FP Yang X Chen YW Fan JG. Steatosis induced CCL5 contributes to early-stage liver fibrosis in nonalcoholic fatty liver disease progress. Trans research: J Lab Clin Med. (2017) 180:103–117.e4. doi: 10.1016/j.trsl.2016.08.006 27639593

[47] Fan Y Zhang W Wei H Sun R Tian Z Chen Y. Hepatic NK cells attenuate fibrosis progression of non-alcoholic steatohepatitis in dependent of CXCL10-mediated recruitment. Liver international: Off J Int Assoc Study Liver. (2020) 40:598–608. doi: 10.1111/liv.14307 31758647

[48] Chu X Jin Q Chen H Wood GC Petrick A Strodel W. CCL20 is up-regulated in non-alcoholic fatty liver disease fibrosis and is produced by hepatic stellate cells in response to fatty acid loading. J Trans Med. (2018) 16:108. doi: 10.1186/s12967-018-1490-y PMC5937820 29690903

[49] Chen J Li X Ge C Min J Wang F. The multifaceted role of ferroptosis in liver disease. Cell Death differentiation. (2022) 29:467–80. doi: 10.1038/s41418-022-00941-0 PMC8901678 35075250

[50] Ma C Han L Zhu Z Heng Pang C Pan G. Mineral metabolism and ferroptosis in non-alcoholic fatty liver diseases. Biochem Pharmacol. (2022) 205:115242. doi: 10.1016/j.bcp.2022.115242 36084708

[51] Li FJ Long HZ Zhou ZW Luo HY Xu SG Gao LC. System X(c) (-)/GSH/GPX4 axis: An important antioxidant system for the ferroptosis in drug-resistant solid tumor therapy. Front Pharmacol. (2022) 13:910292. doi: 10.3389/fphar.2022.910292 36105219 PMC9465090

[52] Lin X Zhang Q Li Q Deng J Shen S Tang M. Upregulation of CoQ shifts ferroptosis dependence from GPX4 to FSP1 in acquired radioresistance. Drug resistance updates: Rev commentaries antimicrobial Anticancer chemotherapy. (2024) 73:101032. doi: 10.1016/j.drup.2023.101032 38198846

[53] Liang D Minikes AM Jiang X. Ferroptosis at the intersection of lipid metabolism and cellular signaling. Mol Cell. (2022) 82:2215–27. doi: 10.1016/j.molcel.2022.03.022 PMC9233073 35390277

[54] Pei J Pan X Wei G Hua Y. Research progress of glutathione peroxidase family (GPX) in redoxidation. Front Pharmacol. (2023) 14:1147414. doi: 10.3389/fphar.2023.1147414 36937839 PMC10017475

[55] Brütsch SH Wang CC Li L Stender H Neziroglu N Richter C. Expression of inactive glutathione peroxidase 4 leads to embryonic lethality, and inactivation of the Alox15 gene does not rescue such knock-in mice. Antioxid Redox Signal. (2015) 22:281–93. doi: 10.1089/ars.2014.5967 25313597

[56] Carlson BA Tobe R Yefremova E Tsuji PA Hoffmann VJ Schweizer U. Glutathione peroxidase 4 and vitamin E cooperatively prevent hepatocellular degeneration. Redox Biol. (2016) 9:22–31. doi: 10.1016/j.redox.2016.05.003 27262435 PMC4900515

[57] Chen J Ou Z Gao T Yang Y Shu A Xu H. Ginkgolide B alleviates oxidative stress and ferroptosis by inhibiting GPX4 ubiquitination to improve diabetic nephropathy. BioMed Pharmacother. (2022) 156:113953. doi: 10.1016/j.biopha.2022.113953 36411664

[58] Zou Y Palte MJ Deik AA Li H Eaton JK Wang W. A GPX4-dependent cancer cell state underlies the clear-cell morphology and confers sensitivity to ferroptosis. Nat Commun. (2019) 10:1617. doi: 10.1038/s41467-019-09277-9 30962421 PMC6453886

[59] Ouyang S Li H Lou L Huang Q Zhang Z Mo J. Inhibition of STAT3-ferroptosis negative regulatory axis suppresses tumor growth and alleviates chemoresistance in gastric cancer. Redox Biol. (2022) 52:102317. doi: 10.1016/j.redox.2022.102317 35483272 PMC9108091

[60] Yang WS SriRamaratnam R Welsch ME Shimada K Skouta R Viswanathan VS. Regulation of ferroptotic cancer cell death by GPX4. Cell. (2014) 156:317–31. doi: 10.1016/j.cell.2013.12.010 PMC4076414 24439385

[61] Liu J Xia X Huang P. xCT: A critical molecule that links cancer metabolism to redox signaling. Mol therapy: J Am Soc Gene Ther. (2020) 28:2358–66. doi: 10.1016/j.ymthe.2020.08.021 PMC7647670 32931751

[62] Margis R Dunand C Teixeira FK Margis-Pinheiro M. Glutathione peroxidase family - an evolutionary overview. FEBS J. (2008) 275:3959–70. doi: 10.1111/j.1742-4658.2008.06542.x 18616466

[63] Gao M Monian P Quadri N Ramasamy R Jiang X. Glutaminolysis and transferrin regulate ferroptosis. Mol Cell. (2015) 59:298–308. doi: 10.1016/j.molcel.2015.06.011 26166707 PMC4506736

[64] Ingold I Berndt C Schmitt S Doll S Poschmann G Buday K. Selenium utilization by GPX4 is required to prevent hydroperoxide-induced ferroptosis. Cell. (2018) 172:409–422.e21. doi: 10.1016/j.cell.2017.11.048 29290465

[65] Forcina GC Dixon SJ. GPX4 at the crossroads of lipid homeostasis and ferroptosis. Proteomics. (2019) 19:e1800311. doi: 10.1002/pmic.201800311 30888116

[66] Qi J Kim JW Zhou Z Lim CW Kim B. Ferroptosis affects the progression of nonalcoholic steatohepatitis via the modulation of lipid peroxidation-mediated cell death in mice. Am J Pathol. (2020) 190:68–81. doi: 10.1016/j.ajpath.2019.09.011 31610178

[67] Zhu Z Zhang Y Huang X Can L Zhao X Wang Y. Thymosin beta 4 alleviates non-alcoholic fatty liver by inhibiting ferroptosis via up-regulation of GPX4. Eur J Pharmacol. (2021) 908:174351. doi: 10.1016/j.ejphar.2021.174351 34280397

[68] Xie Y Kang R Klionsky DJ Tang D. GPX4 in cell death, autophagy, and disease. Autophagy. (2023) 19:2621–38. doi: 10.1080/15548627.2023.2218764 PMC10472888 37272058

[69] Tong J Li D Meng H Sun D Lan X Ni M. Targeting a novel inducible GPX4 alternative isoform to alleviate ferroptosis and treat metabolic-associated fatty liver disease. Acta Pharm Sinica. B. (2022) 12:3650–66. doi: 10.1016/j.apsb.2022.02.003 PMC9513461 36176906

[70] Bersuker K Hendricks JM Li Z Magtanong L Ford B Tang PH. The CoQ oxidoreductase FSP1 acts parallel to GPX4 to inhibit ferroptosis. Nature. (2019) 575:688–92. doi: 10.1038/s41586-019-1705-2 PMC6883167 31634900

[71] Doll S Freitas FP Shah R Aldrovandi M da Silva MC Ingold I. FSP1 is a glutathione-independent ferroptosis suppressor. Nature. (2019) 575:693–8. doi: 10.1038/s41586-019-1707-0 31634899

[72] Dai E Zhang W Cong D Kang R Wang J Tang D. AIFM2 blocks ferroptosis independent of ubiquinol metabolism. Biochem Biophys Res Commun. (2020) 523:966–71. doi: 10.1016/j.bbrc.2020.01.066 31964528

[73] Mao C Liu X Zhang Y Lei G Yan Y Lee H. DHODH-mediated ferroptosis defence is a targetable vulnerability in cancer. Nature. (2021) 593:586–90. doi: 10.1038/s41586-021-03539-7 PMC8895686 33981038

[74] Yoshioka H Kawamura T Muroi M Kondoh Y Honda K Kawatani M. Identification of a small molecule that enhances ferroptosis via inhibition of ferroptosis suppressor protein 1 (FSP1). ACS Chem Biol. (2022) 17:483–91. doi: 10.1021/acschembio.2c00028 35128925

[75] Vietri M Radulovic M Stenmark H. The many functions of ESCRTs. Nat Rev Mol Cell Biol. (2020) 21:25–42. doi: 10.1038/s41580-019-0177-4 31705132

[76] McCullough J Frost A Sundquist WI. Structures, functions, and dynamics of ESCRT-III/vps4 membrane remodeling and fission complexes. Annu Rev Cell Dev Biol. (2018) 34:85–109. doi: 10.1146/annurev-cellbio-100616-060600 30095293 PMC6241870

[77] Dai E Meng L Kang R Wang X Tang D. ESCRT-III-dependent membrane repair blocks ferroptosis. Biochem Biophys Res Commun. (2020) 522:415–21. doi: 10.1016/j.bbrc.2019.11.110 PMC6957708 31761326

[78] NaveenKumar SK Hemshekhar M Kemparaju K Girish KS. Hemin-induced platelet activation and ferroptosis is mediated through ROS-driven proteasomal activity and inflammasome activation: Protection by Melatonin. Biochim Biophys Acta Mol Basis Dis. (2019) 1865:2303–16. doi: 10.1016/j.bbadis.2019.05.009 31102787

[79] Ali SR Parajuli RR Balogun Y Ma Y He H. A nonoxidative electrochemical sensor based on a self-doped polyaniline/carbon nanotube composite for sensitive and selective detection of the neurotransmitter dopamine: A review. Sensors (Basel). (2008) 8:8423–52. doi: 10.3390/s8128423 PMC3791025 27873994

[80] Kim H-L Park YS. Maintenance of cellular tetrahydrobiopterin homeostasis. BMB Rep. (2010) 43:584–92. doi: 10.5483/BMBRep.2010.43.9.584 20846489

[81] Hu Q Wei W Wu D Huang F Li M Li W. Blockade of GCH1/BH4 axis activates ferritinophagy to mitigate the resistance of colorectal cancer to erastin-induced ferroptosis. Front Cell Dev Biol. (2022) 10:810327. doi: 10.3389/fcell.2022.810327 35223839 PMC8866854

[82] Kraft VAN Bezjian CT Pfeiffer S Ringelstetter L Müller C Zandkarimi F. GTP cyclohydrolase 1/tetrahydrobiopterin counteract ferroptosis through lipid remodeling. ACS Cent Sci. (2020) 6:41–53. doi: 10.1021/acscentsci.9b01063 31989025 PMC6978838

[83] Soula M Weber RA Zilka O Alwaseem H La K Yen F. Metabolic determinants of cancer cell sensitivity to canonical ferroptosis inducers. Nat Chem Biol. (2020) 16:1351–60. doi: 10.1038/s41589-020-0613-y PMC8299533 32778843

[84] Yang WS Stockwell BR. Ferroptosis: death by lipid peroxidation. Trends Cell Biol. (2016) 26:165–76. doi: 10.1016/j.tcb.2015.10.014 PMC4764384 26653790

[85] Zou Y Henry WS Ricq EL Graham ET Phadnis VV Maretich P. Plasticity of ether lipids promotes ferroptosis susceptibility and evasion. Nature. (2020) 585:603–8. doi: 10.1038/s41586-020-2732-8 PMC8051864 32939090

[86] Li Z Liao X Hu Y Li M Tang M Zhang S. SLC27A4-mediated selective uptake of mono-unsaturated fatty acids promotes ferroptosis defense in hepatocellular carcinoma. Free Radical Biol Med. (2023) 201:41–54. doi: 10.1016/j.freeradbiomed.2023.03.013 36924851

[87] Kagan VE Mao G Qu F Angeli JP Doll S Croix CS. Oxidized arachidonic and adrenic PEs navigate cells to ferroptosis. Nat Chem Biol. (2017) 13:81–90. doi: 10.1038/nchembio.2238 27842066 PMC5506843

[88] Doll S Proneth B Tyurina YY Panzilius E Kobayashi S Ingold I. ACSL4 dictates ferroptosis sensitivity by shaping cellular lipid composition. Nat Chem Biol. (2017) 13:91–8. doi: 10.1038/nchembio.2239 PMC5610546 27842070

[89] Kagan VE Mao G Qu F Angeli JPF Doll S Croix CS. Oxidized arachidonic and adrenic PEs navigate cells to ferroptosis. Nat Chem Biol. (2017) 13:81–90. doi: 10.1038/nchembio.2238 27842066 PMC5506843

[90] Yang Y Zhu T Wang X Xiong F Hu Z Qiao X. ACSL3 and ACSL4, distinct roles in ferroptosis and cancers. Cancers (Basel) 14. (2022) 14(23):5896. doi: 10.3390/cancers14235896 PMC9739553 36497375

[91] Magtanong L Ko PJ To M Cao JY Forcina GC Tarangelo A. Exogenous monounsaturated fatty acids promote a ferroptosis-resistant cell state. Cell Chem Biol. (2019) 26:420–432.e9. doi: 10.1016/j.chembiol.2018.11.016 30686757 PMC6430697

[92] Tsurusaki S Tsuchiya Y Koumura T Nakasone M Sakamoto T Matsuoka M. Hepatic ferroptosis plays an important role as the trigger for initiating inflammation in nonalcoholic steatohepatitis. Cell Death Dis. (2019) 10:449. doi: 10.1038/s41419-019-1678-y 31209199 PMC6579767

[93] Videla LA Valenzuela R. Perspectives in liver redox imbalance: Toxicological and pharmacological aspects underlying iron overloading, nonalcoholic fatty liver disease, and thyroid hormone action. BioFactors (Oxford England). (2022) 48:400–15. doi: 10.1002/biof.1797 34687092

[94] Day K Seale LA Graham RM Cardoso BR. Selenotranscriptome network in non-alcoholic fatty liver disease. Front Nutr. (2021) 8:744825. doi: 10.3389/fnut.2021.744825 34869521 PMC8635790

[95] Galy B Conrad M Muckenthaler M. Mechanisms controlling cellular and systemic iron homeostasis. Nat Rev Mol Cell Biol. (2023) 25(2):133–155. doi: 10.1038/s41580-023-00648-1 37783783

[96] Crawford DHG Ross DGF Jaskowski LA Burke LJ Britton LJ Musgrave N. Iron depletion attenuates steatosis in a mouse model of non-alcoholic fatty liver disease: Role of iron-dependent pathways. Biochim Biophys Acta Mol basis Dis. (2021) 1867:166142. doi: 10.1016/j.bbadis.2021.166142 33839281

[97] Deng GH Wu CF Li YJ Shi H Zhong WC Hong MK. Caveolin-1 is critical for hepatic iron storage capacity in the development of nonalcoholic fatty liver disease. Military Med Res. (2023) 10:53. doi: 10.1186/s40779-023-00487-3 PMC10631186 37941054

[98] Yu L He M Liu S Dou X Li L Gu N. Fluorescent egg white-based carbon dots as a high-sensitivity iron chelator for the therapy of nonalcoholic fatty liver disease by iron overload in zebrafish. ACS Appl materials interfaces. (2021) 13:54677–89. doi: 10.1021/acsami.1c14674 34756030

[99] Gao H Jin Z Bandyopadhyay G Wang G Zhang D Rocha KCE. Aberrant iron distribution via hepatocyte-stellate cell axis drives liver lipogenesis and fibrosis. Cell Metab. (2022) 34:1201–1213.e5. doi: 10.1016/j.cmet.2022.07.006 35921818 PMC9365100

[100] Li X Wang TX Huang X Li Y Sun T Zang S. Targeting ferroptosis alleviates methionine-choline deficient (MCD)-diet induced NASH by suppressing liver lipotoxicity. Liver international: Off J Int Assoc Study Liver. (2020) 40:1378–94. doi: 10.1111/liv.14428 32145145

[101] Duan J Wang Z Duan R Yang C Zhao R Feng Q. Therapeutic targeting of hepatic ACSL4 ameliorates NASH in mice. Hepatol (Baltimore Md.). (2022) 75:140–53. doi: 10.1002/hep.32148 PMC8688219 34510514

[102] Tong J Lan XT Zhang Z Liu Y Sun DY Wang XJ. Ferroptosis inhibitor liproxstatin-1 alleviates metabolic dysfunction-associated fatty liver disease in mice: potential involvement of PANoptosis. Acta pharmacologica Sin. (2023) 44:1014–28. doi: 10.1038/s41401-022-01010-5 PMC10104837 36323829

[103] Tomitsuka Y Imaeda H Ito H Asou I Ohbayashi M Ishikawa F. Gene deletion of long-chain acyl-CoA synthetase 4 attenuates xenobiotic chemical-induced lung injury via the suppression of lipid peroxidation. Redox Biol. (2023) 66:102850. doi: 10.1016/j.redox.2023.102850 37586249 PMC10450978

[104] He F Zhang P Liu J Wang R Kaufman RJ Yaden BC. ATF4 suppresses hepatocarcinogenesis by inducing SLC7A11 (xCT) to block stress-related ferroptosis. J Hepatol. (2023) 79:362–77. doi: 10.1016/j.jhep.2023.03.016 PMC11332364 36996941

[105] Jiang T Xiao Y Zhou J Luo Z Yu L Liao Q. Arbutin alleviates fatty liver by inhibiting ferroptosis via FTO/SLC7A11 pathway. Redox Biol. (2023) 68:102963. doi: 10.1016/j.redox.2023.102963 37984229 PMC10694775

[106] Cui S Ghai A Deng Y Li S Zhang R Egbulefu C. Identification of hyperoxidized PRDX3 as a ferroptosis marker reveals ferroptotic damage in chronic liver diseases. Mol Cell. (2023) 83:3931–3939.e5. doi: 10.1016/j.molcel.2023.09.025 37863053 PMC10841858

[107] Wang Y Bo J Zhao Z Han Y Zhang Q Liu L. Depletion of Igfbp7 alleviates zebrafish NAFLD progression through inhibiting hepatic ferroptosis. Life Sci. (2023) 332:122086. doi: 10.1016/j.lfs.2023.122086 37714372

[108] Wei S Qiu T Wang N Yao X Jiang L Jia X. Ferroptosis mediated by the interaction between Mfn2 and IREα promotes arsenic-induced nonalcoholic steatohepatitis. Environ Res. (2020) 188:109824. doi: 10.1016/j.envres.2020.109824 32593899

[109] Loomba R Friedman SL Shulman GI. Mechanisms and disease consequences of nonalcoholic fatty liver disease. Cell. (2021) 184:2537–64. doi: 10.1016/j.cell.2021.04.015 PMC12168897 33989548

[110] Zhuge A Li S Yuan Y Han S Xia J Wang Q. Microbiota-induced lipid peroxidation impairs obeticholic acid-mediated antifibrotic effect towards nonalcoholic steatohepatitis in mice. Redox Biol. (2023) 59:102582. doi: 10.1016/j.redox.2022.102582 36584600 PMC9830314

[111] Febbraio MA Reibe S Shalapour S Ooi GJ Watt MJ Karin M. Preclinical models for studying NASH-driven HCC: how useful are they? Cell Metab. (2019) 29:18–26. doi: 10.1016/j.cmet.2018.10.012 30449681 PMC6326872

[112] Fan B Cheng C Yang Y Wang P Xia H Wu M. Construction of an adverse outcome pathway framework based on integrated data to evaluate arsenic-induced non-alcoholic fatty liver disease. Environ Int. (2024) 183:108381. doi: 10.1016/j.envint.2023.108381 38118209

[113] Corradini E Buzzetti E Dongiovanni P Scarlini S Caleffi A Pelusi S. Ceruloplasmin gene variants are associated with hyperferritinemia and increased liver iron in patients with NAFLD. J Hepatol. (2021) 75:506–13. doi: 10.1016/j.jhep.2021.03.014 33774058

[114] Chitapanarux T Tienboon P Pojchamarnwiputh S Leelarungrayub D. Open-labeled pilot study of cysteine-rich whey protein isolate supplementation for nonalcoholic steatohepatitis patients. J Gastroenterol Hepatol. (2009) 24:1045–50. doi: 10.1111/j.1440-1746.2009.05865.x 19638084

[115] Lv T Fan X He C Zhu S Xiong X Yan W. SLC7A11-ROS/αKG-AMPK axis regulates liver inflammation through mitophagy and impairs liver fibrosis and NASH progression. Redox Biol. (2024) 72:103159. doi: 10.1016/j.redox.2024.103159 38642501 PMC11047786

[116] Keam SJ. Resmetirom: first approval. Drugs. (2024) 84(6):729–735. doi: 10.1007/s40265-024-02045-0 38771485

[117] Zhang L Dai X Wang L Cai J Shen J Shen Y. Iron overload accelerated lipid metabolism disorder and liver injury in rats with non-alcoholic fatty liver disease. Front Nutr. (2022) 9:961892. doi: 10.3389/fnut.2022.961892 36304234 PMC9593083

